# Engaging LGBTQ+ Youth in Human-Centered Design of a Digital Health Intervention via Discord: Implementation Case Study

**DOI:** 10.2196/80852

**Published:** 2026-03-30

**Authors:** Jacob Gordon, Julianna Lorenzo, Andrés Alvarado Avila, Bryant Norton, Eva Minahan, George J Greene, Ariel W Halle, Kathryn Macapagal

**Affiliations:** 1 College of Nursing University of Cincinnati Cincinnati, OH United States; 2 Impact Institute Department of Medical Social Sciences Northwestern University Chicago, IL United States; 3 Medical Social Sciences Feinberg School of Medicine Northwestern University Chicago, IL United States; 4 Feinberg School of Medicine Northwestern University Chicago, IL United States

**Keywords:** sexual and gender minority groups, LGBTQ+, human-centered design, Discord, social media

## Abstract

**Background:**

Lesbian, gay, bisexual, transgender, and queer/questioning, plus (LGBTQ+) youth experience significant health challenges relative to their peers, including higher rates of HIV, sexually transmitted infections, and mental health symptoms, partly due to minority stressors. Digital health interventions hold promise for addressing these issues, but their effectiveness hinges on human-centered co-design that ensures relevance and engagement.

**Objective:**

This study aimed to examine the use of Discord as a platform for conducting human-centered design (HCD) activities to adapt a digital text-based intervention designed to improve HIV testing rates among LGBTQ+ youth.

**Methods:**

We recruited 21 LGBTQ+ youth (aged 13-18 years) in the United States via social media and participant registries, oversampling minoritized gender, racial, and ethnic identities to ensure diverse representation. Over 9 months, participants engaged in structured HCD activities on a private Discord server, including polls, open-ended discussions, and interactive feedback tasks. Design insights were collected iteratively and used to refine the intervention in real time. We also surveyed participants to examine the acceptability of Discord as a tool for hosting the HCD process.

**Results:**

We identified best practices for integrating HCD methods within Discord, including cocreating the server environment with participants and enabling real-time iteration of intervention components based on youth input. The privacy of the Discord server supported psychological safety; facilitated open and effective communication between participants and the research team; and fostered an informal, familiar atmosphere.

**Conclusions:**

Discord provides an effective and acceptable environment for conducting HCD processes in the design of digital health interventions. Its structural features, including anonymity, accessibility, and community-driven interaction, facilitated meaningful youth engagement in co-design activities. These insights offer a model for leveraging social media platforms to support participatory intervention development for LGBTQ+ populations.

## Introduction

Lesbian, gay, bisexual, transgender, and queer/questioning, plus (LGBTQ+) youth in the United States face many health challenges, including disproportionately high rates of HIV and poor mental health compared to their cisgender heterosexual peers [1-4]. LGBTQ+ youth’s risks are further heightened if they hold multiple minoritized identities, including sexual orientation, gender, race, and ethnicity, and these risks often stem from minoritized stress experiences (ie, stigma and harassment) [5-7]. However, existing interventions are often designed without considering the unique needs or experiences of LGBTQ+ youth, which may undermine intervention effectiveness.

Digital interventions can provide LGBTQ+ youth with accessible and equitable interventions and information to tackle low rates of HIV testing and uptake of HIV or sexually transmitted infection prevention tools, minority stressors, and poor mental health outcomes [8-10]. Indeed, most adolescents from various racial and economic backgrounds access the internet daily [11]. Furthermore, texting remains the dominant mode of communication for adolescents [12]. Although digital interventions hold promise for improving youth health outcomes, this technology alone does not guarantee success. It is essential that digital interventions are engaging, evidence based, and acceptable for diverse LGBTQ+ end users [9]. One method to ensure these qualities in digital interventions is by using a human-centered design (HCD) approach with LGBTQ+ youth for the development and implementation of interventions [13,14].

HCD incorporates user feedback throughout the intervention design and implementation process, contributing to a more acceptable and usable final digital health intervention. Co-design is one strategy that can be used within HCD that purposefully includes end users in the development process [15-17]. Together, the HCD approach has been shown to be feasible with LGBTQ+ youth in both online and in-person environments for the cocreation of digital interventions that target health disparities among LGBTQ+ youth. These include interventions that improve outcomes such as loneliness, depressive symptoms, and relationship abuse [13,18,19].

Discord (Discord Inc) is a social media platform that has great potential to accommodate the HCD process with LGBTQ+ youth. Discord is a communication platform that allows users to create servers dedicated to specific interests or communities in which people can communicate. Discord’s ability to foster communities and facilitate interaction has contributed to its widespread use among LGBTQ+ users, who can access social support and resources through the platform [20]. LGBTQ+ youth may use Discord partly due to its privacy features, which allow these groups to develop in relative comfort and safety, for example, by enabling users to remain anonymous or to create private servers that help keep out bad actors [21].

Previous research has used Discord for research purposes, such as eliciting experiences of digital interventions, recruitment, hosting design workshops, and forming advisory committees, all of which have shown promise for engaging LGBTQ+ youth [22-25]. However, the use of Discord as a holistic setting that can support all of these research purposes has not been explored to our knowledge. This study describes the use of Discord as an online setting to engage LGBTQ+ youth in the HCD process for the development and adaptation of an existing digital intervention and examines its acceptability from the user perspective. As social media platforms often fall out of favor with adolescents, this paper presents best practices for engaging youth in HCD methods that can be used on Discord and across different social media platforms.

## Methods

### Background

The following activities occurred in the context of a hybrid effectiveness-implementation study, which first sought to adapt and modernize a previous sexual health intervention called Guy2Guy, originally developed from 2012 to 2013 [26]. Guy2Guy was intended to increase condom use and other HIV prevention and testing behaviors among gay and bisexual teenage boys. The intervention consisted of 8 to 10 daily texts sent to participants over 6 weeks and included various interactive features, including a chatbot and one-on-one anonymous texting buddies. This intervention was associated with a 3-fold increase in HIV testing in the active treatment arm vs an information-only control arm [27,28].

In the new study, which began a decade after Guy2Guy’s original development, we sought to expand the intervention to be inclusive of LGBTQ+ adolescents who could benefit from HIV prevention and testing behaviors, adapt Guy2Guy to include updated HIV and sexually transmitted infection prevention information, modernize the texting delivery (eg, multimedia messaging service rather than SMS), and update other technological components such as the chatbot and texting buddies. This adaptation and modernization process was informed by feedback from a diverse sample of LGBTQ+ youth (aged 13-18 years; n=21) hosted on Discord over a 9-month period.

### Recruitment

We recruited participants through paid social media advertisements and an existing institute-wide participant registry. Social media advertisements were developed following the CAN-DO-IT (conceptualize scope, acquire expertise, navigate platform and strategy, develop ad content, optimize recruitment workflow, implement campaign, track, and respond) model [28] and were posted on Meta platforms (Instagram and Facebook). Eligibility criteria were as follows: (1) sexual and/or gender minority identity; (2) age 13 to 18 years; (3) ability to read English at an eighth-grade level; (4) for minors, ability to provide informed assent and pass a capacity to consent assessment; and (5) residence in the United States or US territories.

Interested individuals were directed to complete the eligibility screening, consent, and capacity to consent surveys in Research Electronic Data Capture (REDCap; Vanderbilt University). We purposely oversampled transgender, gender-diverse, and minoritized racial and ethnic participants to ensure diverse feedback. A total of 24 participants were enrolled in the study. Only 12.5% (3/24) of participants formally requested to withdraw before the Discord server officially started its research activities, most commonly citing competing time commitments. Over the active course of the 9-month period of Discord activities (March 2023 to November 2023), no additional participants withdrew. The remaining 87.5% (n=21) of active participants comprised the analytic sample for this paper and participated in the survey reported here, as well as the primary study activities described later.

### Ethical Considerations

This study was approved by the Northwestern University Institutional Review Board (IRB; STU00217358 and STU00222652). Informed consent was obtained from all participants prior to participation in the advisory council. A waiver of parental permission was approved by the IRB on the basis of minimal risk and the potential for increased harm if parental consent were required. Previous IRB-approved studies conducted by the investigative team with sexual and gender minority (SGM) adolescents younger than 18 years have demonstrated that parental permission does not consistently function as a protective mechanism for this population [29]. Many SGM adolescents are not out to their parents and do not disclose sexual orientation, gender identity, or sexual behavior due to fear of rejection, victimization, or family conflict [29]. Therefore, requiring parental consent could increase risk by inadvertently “outing” participants or exposing them to harm and would likely result in systematic exclusion of youth most in need of sexual health research. Consistent with previous studies of adolescent SGM sexual health, the IRB determined that requiring parental permission would introduce significant sampling bias and may compromise participant welfare, while the study procedures themselves posed no greater than minimal risk [30-33]. Assent procedures, confidentiality protections, and access to supportive resources were implemented to ensure ethical participation. Through the consent process and subsequent orientation, participants were given information about the activities associated with the advisory council, details concerning privacy and confidentiality, possible risks and benefits, and reminders that participation was voluntary and could be stopped at any time.

We paid participants US $30 per month for active participation, defined as answering 75% or more of the questions asked in each month, delivered via virtual gift cards. Participants also had the opportunity to get paid an additional US $60 every 3 months for completing optional extra activities, such as creating a social media post or providing more in-depth feedback about the intervention. All data were stored on private servers accessible only to study staff, in compliance with the Health Insurance Portability and Accountability Act. Participants were assigned unique identifiers for analysis and reporting requirements. All data were deidentified, and all personally identifying information was removed before data were shared beyond the research team to ensure privacy and confidentiality.

### Measurement

High-level feedback about the acceptability of Discord for these activities was assessed by an online survey completed by all participants (21/21, 100%) hosted through Qualtrics (Qualtrics Inc) 6 months after the start of Discord activities. Questions were developed for the purpose of understanding perceived acceptability in the context of the features of Discord (eg, “I felt comfortable sharing my thoughts and ideas on Discord”) and were aligned with the core principles of HCD, such as reporting iterative changes based on participant feedback (eg, “I felt like the research team echoed or reported our SHER [Sharing Health Educational Resources] feedback back to us after making changes”).

Additionally, we allowed participants to provide open-ended answers to the following question: “Would you use Discord again for future research projects like this? Please tell us why.” Results of this survey and the open-ended answers are presented together in thematic groupings and include the themes of psychological safety and ease of communication. We used a rapid, inductive qualitative approach to analyze open-ended Discord feedback [34,35]. Two team members independently reviewed and grouped responses, refining categories through discussion in weekly meetings. Because the purpose was exploratory and aligned with HCD processes, we resolved differences by consensus rather than by computing interrater reliability. Coding and data management were conducted in Microsoft Excel.

### Discord Configuration and Process Overview

The study’s Discord server consisted of multiple channels organized into 3 categories: “Information,” “Activities,” and “Creative and Fun.” “Information” channels included server guidelines, resources, a channel to directly contact staff, and a copy of the consent form. “Activities” channels consisted of weekly questions that engaged participants in the HCD process. Feedback from the HCD activities was collected through poll responses and open-ended threads on Discord. When responding to poll questions, participants were prompted to react to staff messages by selecting the emoji corresponding to their chosen vote, and open-ended questions used the “thread” feature to organize responses in one location within the channel.

All weekly questions, extra activities, and retention activities on Discord were developed during weekly collaborative staff meetings with the full study team. Staff identified “themes” for weekly questions and key areas for participant feedback based on the needs of the project (eg, new intervention content, adaptation of existing intervention content, and software design and development). The questions and relevant materials were organized in an Excel document. Discord moderation team members then reviewed the questions proposed by the research team to ensure consistency with the language and tone of the Discord server. After internal approval, questions were posted to Discord by research staff. Participant feedback was copied and pasted into a separate Excel document to facilitate easier visualization and data analysis. Key findings from participants were presented during weekly research team meetings and content development meetings, where the research team decided which feedback to implement. This process included qualitatively interpreting Discord posts through these team discussions to contextualize participant comments and clarify emerging insights. Any clarifying questions were also proposed back to the participants.

### Study Design and Theoretical Framework

#### Overview

This study used an HCD approach to engage LGBTQ+ youth as cocreators in adapting a digital health intervention. HCD emphasizes an iterative approach to design that can be applied under the EDIPT (emphasize, define, iterate, prototype, and test) model with end users to ensure solutions are grounded in lived experience and are usable [36]. Our approach was also informed by participatory and co-design methods, which center on user agency and position participants as active contributors to the design process [37]. We drew on design justice principles (eg, equitable and inclusive solutions), emphasized community-led design, and adopted an intentional approach to intersectional differences by diversifying recruitment.

Discord was selected as the design space for its cultural relevance, familiarity, and flexibility for LGBTQ+ youth and based on recommendations from the research team’s youth advisors in a previous study [38]. Its hybrid structure, which included supporting both asynchronous and synchronous interaction, allowed for accessible, sustained engagement in a setting aligned with youth preferences and safety needs. We describe the HCD process by presenting a polling feature that was co-designed with participants in this study.

#### Empathize: Building Trust and Understanding Youth Needs

We began by creating a welcoming, private Discord server designed with input from LGBTQ+ youth advisors to ensure safety and cultural relevance. The approximate weeks and months associated with each phase, along with an estimate of moderation activities, are presented in Table 1.

**Figure 1:**
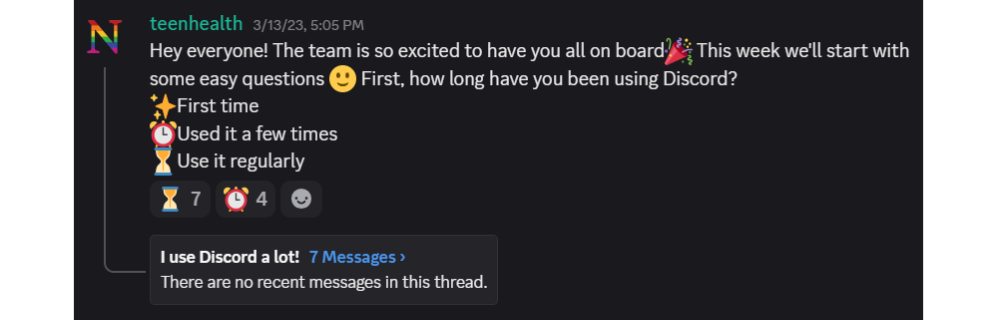
Screenshot of the moderation team using an icebreaker activity on Discord to engage participants.

In the first month, moderators heavily focused on orientation and empathizing with participants through low-effort onboarding prompts (eg, introductions, pronoun sharing, community agreements, and icebreaker questions). However, we continued this stage throughout the entirety of the design process. These activities generated active open-ended threads monthly and served as early relationship-building activities (Figure 1). When appropriate, moderators responded to each post to model engagement and build trust throughout the length of participation. Participants also began sharing early experiences with digital interventions, which helped us understand their baseline needs and expectations.

**Table 1:** EDIPT (emphasize, define, iterate, prototype, and test) design activities timeline.

EDIPT phase	Months engaged	Weeks engaged	Polls	Open-ended prompts
Empathize	1-9	All weeks	1-2 per week^a^	1-2 per week^a^
Define	1-9	2, 3, 5-7, 9, 15, 21, and 34	4-6 per week	2-4 per week
Ideate	1-9	3-5, 8, 9, 19, 21, 22, and 35	4-5 per week	2-4 per week
Prototype	2-9	7, 8, 12, 23, and 35	3-5 per week	2-3 per week
Test	1-6 and 9	4, 11, 13, 14, and 23-25	4-6 per week	2 per week

^a^Empathize phase had a higher frequency of Discord engagement closer to orientation.

#### Define: Cocreating Problem Statements

**Figure 2:**
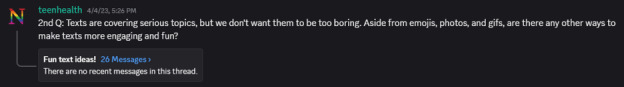
Screenshot of the moderation team asking participants to answer a prompt about making the digital intervention more engaging.

Using guided prompts in dedicated threads and polls, participants identified challenges they faced with text-based interventions. Participants offered examples of what felt disengaging or ineffective. Figure 2 shows one of these open-ended discussion threads. The research team synthesized these contributions into draft problem statements and posted them back to the group for refinement. In this example, this iterative exchange clarified that one major challenge was that text-based interventions felt “boring” or insufficiently interactive. This insight directly informed the next design phase.

#### Ideate: Brainstorming Youth-Led Solutions

**Figure 3:**
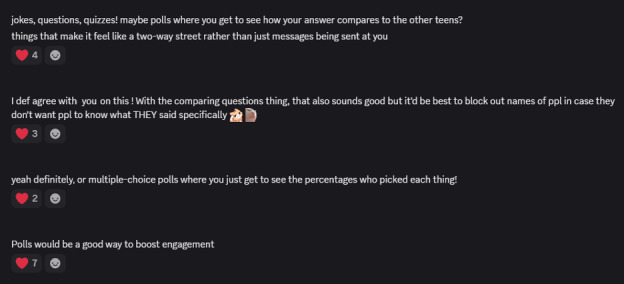
Screenshot of participant-generated solutions for improving engagement in the digital intervention.

We facilitated a series of ideation threads in which participants proposed features that they believed would improve engagement to the problem they specified. Youth generated solution ideas across multiple threads, using reactions and emoji-voting features to identify those with the highest level of interest, as shown in Figure 3. For example, participants prioritized adding quizzes and polls as a way to increase interactivity. These ideation artifacts (eg, thread transcripts, prioritized lists, and voted-on features) formed the basis of the initial mock-ups that the research team crafted.

#### Prototype: Exploring Early Concepts Together

**Figure 4:**
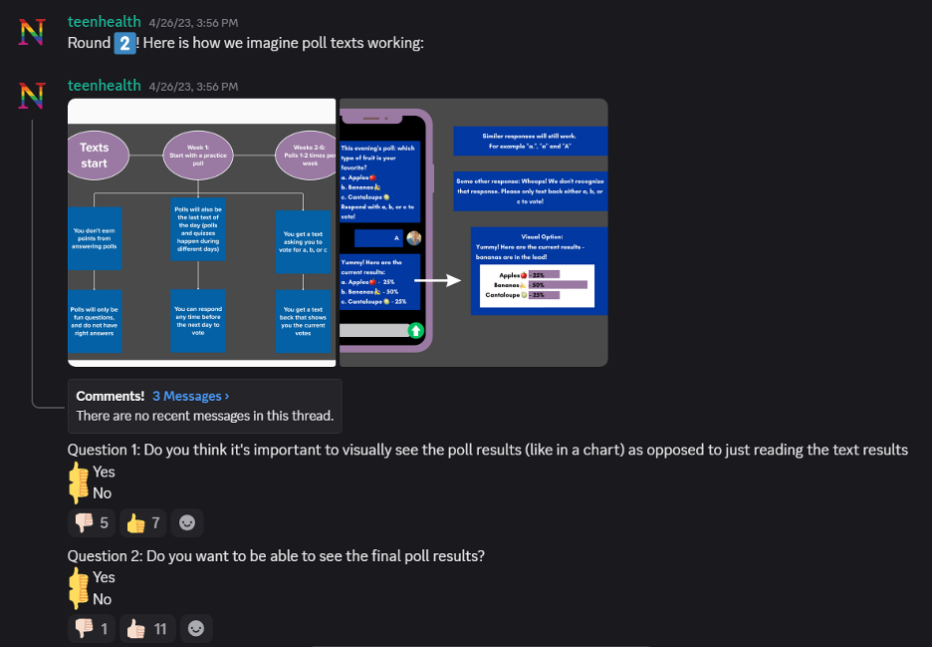
Screenshot of the moderation team showing participants prototype mock-ups of their ideas on Discord.

The research team translated youth-generated ideas into low-fidelity prototypes using Canva, which we introduced in Discord as static screenshots. Participants evaluated these prototypes in dedicated threads and polls, responding weekly to specific prompts to further iterate on the prototype (eg, preferred visual formats, clarity of instructions, and color preferences), as shown in Figure 4. Their feedback informed 3 rounds of prototype refinement. Updated mock-ups were posted back to the server after each cycle, creating a clear, traceable artifact trail documenting iterative changes.

#### Test: Gathering Feedback and Refining Ideas

**Figure 5:**
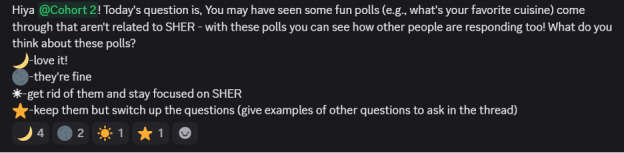
Screenshot of the moderation team gathering participant feedback on the prototype engagement strategies that were used in alpha testing on Discord. SHER: Sharing Health Educational Resources.

Testing was done throughout the design process, starting with simple 1-day testing sessions during which we manually sent texts to select participants early in the process. Participants also engaged in informal alpha testing when the early poll features were built into the intervention during the last weeks of the Discord process. Using Discord polls and open discussion prompts, youth shared their experiences with the prototype poll feature after participating in the test. These discussions generated additional refinements to the poll prototype, as shown in Figure 5. Finalized prototype features (Multimedia Appendix 1) directly reflect this testing feedback. Across the 9-month engagement period, moderators posted design prompts approximately weekly, resulting in more than 40 active threads spanning the EDIPT phases. These artifacts, including threads, refined problem statements, ideation lists, prototype screenshots, and feedback discussions, demonstrate iterative, youth-driven engagement throughout the process.

## Results

### Overview

Full sociodemographic information of the participants is presented in Table 2. Participants had a mean age of 17.4 (SD 0.98) years. The sample was diverse across multiple identities, including sexual orientation, gender, and location, with participants representing 16 different states. Results of the survey informed our best practices when engaging in HCD on Discord for digital intervention design and adaptations. For the full results of the survey, refer to Multimedia Appendix 2.

**Table 2:** Sociodemographic variables (N=21).

Sociodemographic variables			Participants, n (%)
**Age (years)**			
	15	1 (4.8)	
	16	2 (9.5)	
	17	7 (33.3)	
	18	9 (42.9)	
	19	2 (9.5)	
**Gender**			
	Man or boy	16 (76.2)	
	Woman or girl	4 (19.0)	
	Gender nonconforming	1 (4.8)	
**Race**			
	American Indian or Alaska Native	4 (19.0)	
	Black or African American	3 (14.3)	
	Native Hawaiian or other Pacific Islander	1 (4.8)	
	Other	3 (14.3)	
	White	10 (47.6)	
**Transgender identity**			
	Yes	9 (42.9)	
	No	11 (52.4)	
	Unsure	1 (4.8)	
**Sexual orientation**			
	Gay	6 (28.6)	
	Bisexual or pansexual	8 (38.1)	
	Queer	4 (19.0)	
	Questioning	2 (9.5)	
	Other	1 (4.8)	

### Discord Facilitated Psychological Safety and Familiarity

One benefit of using Discord for HCD activities was the ease of participation and the ability to ensure safety and anonymity. Participants consistently described feeling at ease participating in design activities within Discord compared to more formal research settings, which likely contributed to high engagement. One reason for this was that participants largely reported no concerns about their privacy on Discord; about 90% (19/21) responded that they had no privacy concerns on the platform. The flexibility to engage asynchronously, use chosen names or pronouns, and opt into different modes of participation contributed to high engagement across our HCD activities. In total, 80% (n=17) of participants responded that they were comfortable visiting Discord to engage in the HCD activities and posting their opinions in threads and activities online. Additionally, participants reported that their familiarity with Discord in their personal lives made them likely to use the platform again for a similar project. Two participants mentioned this factor:

It’s convenient because I am active on Discord for other purposes, and I am able to stay updated with the server due to my frequent use of the platform.

I primarily use Discord anyway and it’s very helpful for this.

### Discord Maintained Effective Communication Between the Research Team and Participants

A critical component of the HCD process is maintaining effective, ongoing communication between design teams and participants across the EDIPT phases. We found that Discord facilitated highly efficient communication among participants, moderators, and the research team. Most participants (18/21, 85%) reported feeling that the research team listened to their suggestions for improving the intervention, and 90% (n=19) noted that the team communicated how their feedback directly influenced design changes. This feedback loop was essential; participants overwhelmingly indicated that seeing their ideas reflected in the evolving intervention strengthened their sense of contribution and improved their overall experience with the HCD process. Participants reiterated this qualitatively:

I think it’s pretty easy to use and works well for communicating with others and the research team.

It is easier to speak with other participants and researchers than email and text.

Improved communication between the research team and participants may have also supported conflict resolution. Although this was not assessed in the survey, a participant reported a conflict directly to the research team, suggesting a level of trust in the moderation process.

### HCD Was Most Effective in Casual, Low-Stakes Formats

Lightweight tools such as emoji reactions and informal polls proved to be more engaging and generative than structured surveys or longer text-based threads. Participants (19/21, 90%) agreed that it was easy to share feedback on Discord using our methods, which included using preselected emojis to respond to questions (Figure 1). Additionally, a majority of participants (n=16, 76%) mentioned that they felt they were able to share all their suggestions for the prototypes and interventions, which suggests that our lightweight approach to data collection was acceptable and capable of capturing their feedback. For instance, one participant mentioned the following:

It was easy to use [which] makes participation simple.

Another participant commented about the tone and the use of emojis in the activities:

I think it simplifies data gathering by putting it into a familiar text format.

To establish best practices for using Discord for HCD design purposes, the moderation team described effective, casual study procedures that improved engagement and psychological safety. Refer to Textbox 1 for a brief report on the best practices reported by the moderation team.

**Best moderation practices for improving engagement and psychological safety on Discord.**

**Procedures that improved engagement and psychological safety**

- Conduct a group orientation for Discord to ensure participants understand how Discord works and to set up first connections between moderators and participants.
- Include strict rules of conduct in the orientation that participants must agree to abide by to encourage respectful behavior.
- Build trust between moderators and participants by purposefully finding opportunities to bond, such as through shared interests and introductions, within the same day in nonresearch channels.
- Ensure that the moderation team responds daily to what participants are posting by responding with comments and/or emojis.
- Avoid extended pauses in content.
- Use emojis to collect data and limit the number of open-ended questions so participants can respond quickly. If more detailed responses are needed, communicate this clearly.
- Communicate how participant feedback has impacted the intervention content and design. If feedback is not used, explain why.
- Allow flexibility for participants to take breaks or choose between different activities.
- Conduct ongoing assessments of participation burden in relation to the compensation offered.

## Discussion

### Principal Findings

Our team developed a novel research participation hub on Discord to engage LGBTQ+ youth in HCD for the adaptation of a digital health intervention. Our methods encouraged high engagement and, most importantly, were considered acceptable by participants. Hosting this process on Discord allowed direct and instantaneous access to LGBTQ+ youth for supporting the successful adaptation of a digital health intervention component. Our findings support the acceptability of Discord as a tool for engaging in HCD activities in a community-based participatory research in a digital setting. We demonstrated how this digital environment can facilitate the co-design of digital health interventions.

The moderation team also assessed satisfaction, Discord acceptability, and Discord server recommendations with participants quarterly. For instance, when we assessed participant suggestions for improving data collection on Discord, participants mentioned that it was difficult to respond to specific prompts (Multimedia Appendix 3). We changed our Discord structure so that all posts contained all possible response emojis. This way, participants could click on the emoji reaction to “vote” without manually searching for the specific emoji. By asking for feedback surrounding the design of data collection itself, participants can cocreate and democratize the server environment to meet their own needs. This approach also ensures that researchers can improve participant retention, satisfaction, and participation.

There are many benefits to using Discord in the HCD process for digital interventions. Discord allowed for substantial anonymity (eg, not requiring real names or profile pictures), does not require transit, and does not require parental or guardian approval for participation among users older than 13 years. In addition, the platform is familiar and easy to navigate for many young people in the United States, particularly LGBTQ+ youth, who are often highly connected in digital communities. Our participants reported that the HCD activities conducted on Discord were widely acceptable. Additionally, Discord allowed affordances that followed the principles of community-based participatory research, specifically shared decision-making, mutual ownership of the research process and its outcomes, colearning, and recognition of the community as a unit of identity [37,39].

We also found that the digital affordances and structure of Discord were useful for this research. We were able to sort different conversations into specific channels for specific topics. For example, we had separate channels for more HCD-focused discussions, and others focused on building a sense of community. This fostered a more holistic and inclusive research environment for participants, where they could be present on Discord not only as study participants helping to design a health intervention but as individuals with diverse interests who were invested in being part of a community [39-41]. The use of Discord for engaging in HCD activities is effective at lowering barriers between academic professionals and LGBTQ+ youth [42] and offers avenues for direct and clear communication between the groups [21]. By centering the design of interactions around meeting youth on platforms they already use, such as Discord, researchers are able to develop bonds with and learn more efficiently from community youth, leading to improved intervention design. Liem et al [43] also demonstrated the process of how the feedback from this Discord youth advisory board informed the adaptation of a separate digital health intervention.

### Limitations and Strengths

Social media platforms may fall in and out of favor over time, so adapting digital HCD methods as existing platforms evolve and new platforms arise will be necessary. However, the best practices we shared are generalizable to many current social media platforms that researchers may use for the HCD of digital interventions with youth, LGBTQ+ youth, and others. While an HCD approach to digital interventions is valuable, there are clear limitations. Our own study relied on a small number of end users, which, while common in HCD work, does hamper our ability to generalize to other end users [44]. Sample adequacy in HCD is instead evaluated based on the diversity of perspectives, sufficiency of idea generation, and whether additional participants yield novel design insights. Across sessions, we observed recurring themes and did not observe substantively new insights emerging, indicating that the sample size was appropriate for the aims of this study.

Our purposeful sampling approach also helped to ensure a diversity of perspectives by intentional recruitment efforts focused on racial and ethnic minority persons who are LGBTQ+. Consequently, nearly half of the sample identified as belonging to racial or ethnic groups other than White. In addition to sample size, participation required access to Discord and a baseline level of digital literacy, which may have deterred some youth and introduced platform-specific selection bias. These factors limit the generalizability of our accessibility model. However, participants in this study demonstrated high familiarity with Discord, consistent with previous research showing higher Discord use among LGBTQ+ youth than among heterosexual peers and broader patterns of youth social media engagement [20,45]. Future work should assess variation in digital literacy and examine whether these methods translate to other platforms.

In addition to sample size, participation required access to Discord and a basic level of digital literacy, which may have excluded or deterred some youth and introduced platform-specific bias. These barriers limit the generalizability of our “accessible” model. Understanding the extent of youth digital literacy and whether the reported methods are usable on other platforms should be addressed in future work.

Furthermore, as HCD is an iterative process, it can be infeasible for some intervention developers due to the cost and time required for maintaining an ongoing Discord server for the purpose of intervention design and adaptation. Our 9-month Discord engagement period was moderately resource intensive and may not be easily scalable. However, while certain routine tasks could be automated (eg, onboarding or reminder bots), maintaining participant trust required active human facilitation. Moderation from research staff would have to be responsive to a higher number of participants in any one server. Future research should identify which components can be automated and explore the most effective amount of moderation. Despite this potential limitation, this online methodology was cost-effective and potentially more feasible for youth participation than intensive in-person alternatives, such as iterative focus groups, which require travel expenses and logistics. While other studies have used Discord for similar research purposes, they often used already existing Discord servers or communities [25]. Our approach allowed for the bottom-up and community-forward creation of a Discord server that fostered participatory benefits and improved engagement throughout the HCD process.

### Conclusions

This study highlights the innovative use of Discord as a dynamic, youth-friendly platform for engaging LGBTQ+ youth in HCD activities. By leveraging a space where participants already feel comfortable and connected, we demonstrate how this digital environment can facilitate more authentic, accessible, and sustained involvement in the co-design of digital health interventions. Our findings underscore the potential of integrating familiar social media technologies into participatory research practices, ultimately advancing inclusive and acceptable digital intervention design. This work contributes valuable insights for researchers and designers who engage in HCD to create more equitable and responsive interventions for marginalized youth communities.

## Appendix

Multimedia Appendix 1 Screenshots of polling feature prototypes in alpha testing.

Multimedia Appendix 2 Table showing full acceptability survey.

Multimedia Appendix 3 Screenshot of participant feedback on improving data collection on Discord.

## Abbreviations

CAN-DO-IT: conceptualize scope, acquire expertise, navigate platform and strategy, develop ad content, optimize recruitment workflow, implement campaign, track, and respond
EDIPT: emphasize, define, iterate, prototype, and test
HCD: human-centered design
IRB: Institutional Review Board
LGBTQ+: lesbian, gay, bisexual, transgender, and queer/questioning
REDCap: Research Electronic Data Capture
SGM: sexual and gender minority
SHER: Sharing Health Educational Resources

## Data Availability

The datasets generated or analyzed during this study are not publicly available due to the ongoing intervention but are available from the corresponding author on reasonable request.
