# Supplementary figures and images for: Engaging LGBTQ+ Youth in Human-Centered Design of a Digital Health Intervention via Discord: Implementation Case Study
Source: JMIR Form Res. 2026 Mar 30;10:e80852. doi: 10.2196/80852 (PMC13077274; doi:10.2196/80852)

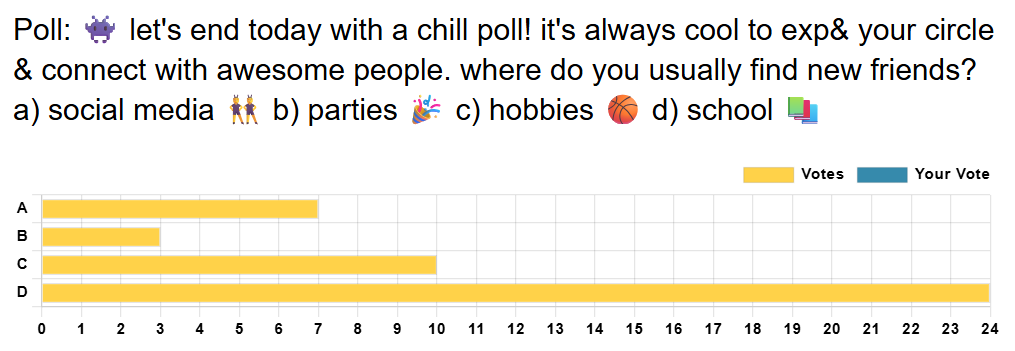


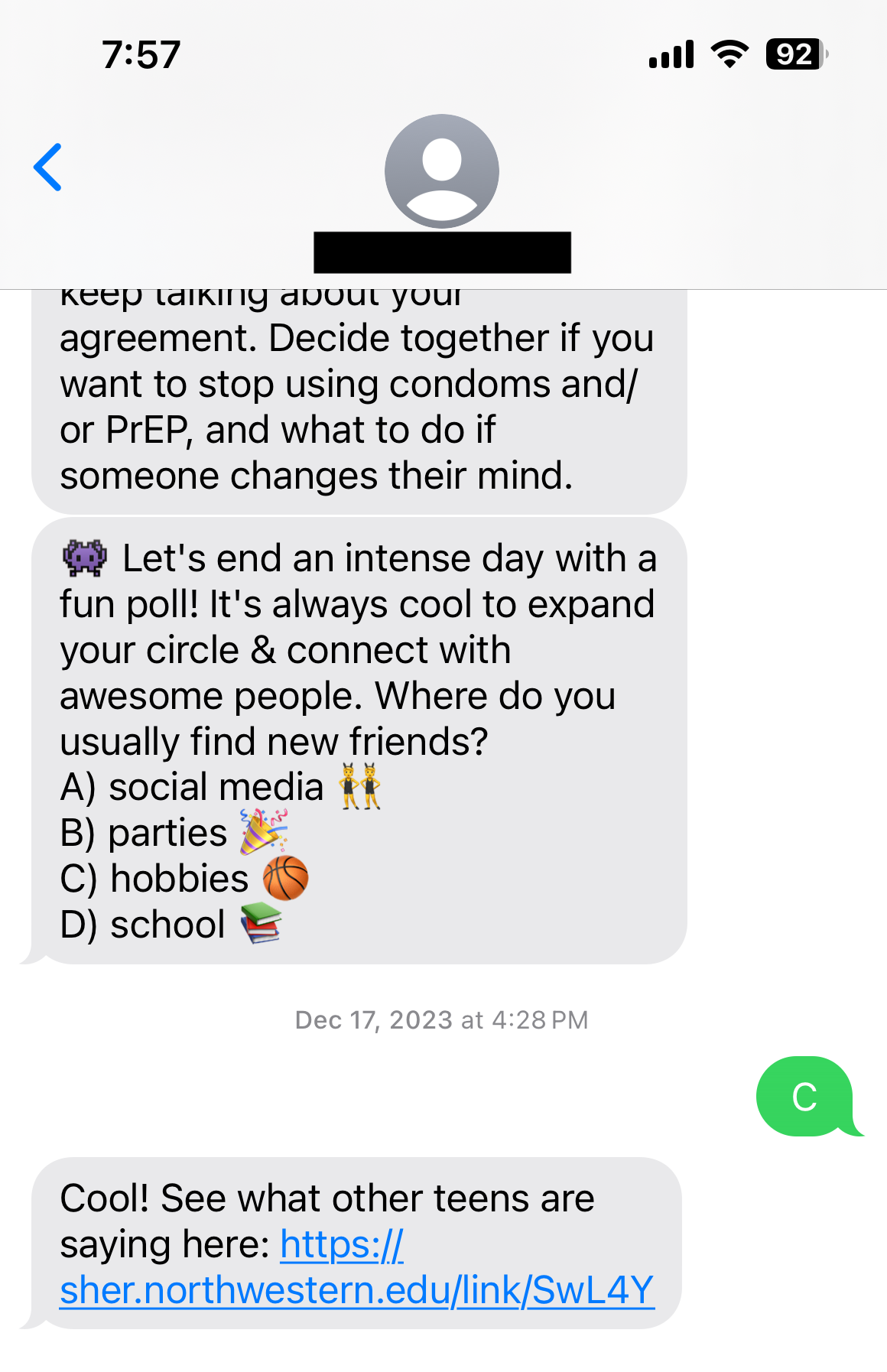

Supplement: Multimedia Appendix 1 [file formative_v10i1e80852_app1.docx]

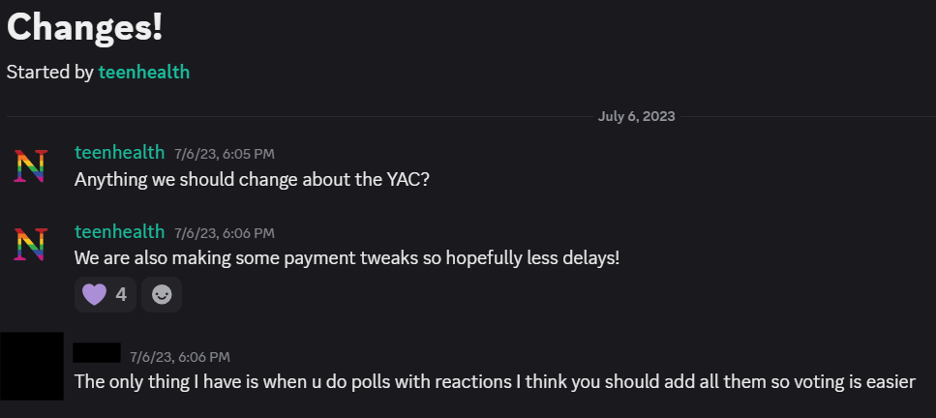

Supplement: Multimedia Appendix 3 [file formative_v10i1e80852_app3.png]
