# Supplementary material for: Engaging LGBTQ+ Youth in Human-Centered Design of a Digital Health Intervention via Discord: Implementation Case Study
Source: JMIR Form Res. 2026 Mar 30;10:e80852. doi: 10.2196/80852 (PMC13077274; doi:10.2196/80852)
Supplement: Multimedia Appendix 2 [file formative_v10i1e80852_app2.pdf]

HCD survey items	Strongly disagree	Somewhat disagree	Neither agree nor disagree	Somewhat agree	Strongly agree
Discord was easy to use for communicating my suggestions for improving SHER	1 (5%)	1 (5%)	0 (0%)	2 (10%)	16 (80%)
I felt like the research team echoed/reported our SHER feedback back to us after making changes	1 (5%)	0 (0%)	1 (5%)	4 (20%)	14 (70%)
I liked using discord to provide suggestions for improving SHER	2 (10%)	1 (5%)	0 (0%)	2 (10%)	15 (75%)
I was concerned about my privacy on the Discord	13 (65%)	5 (25%)	0 (0%)	1 (5%)	1 (5%)
I found it easy to talk to others, and our moderators on Discord	1 (5%)	0 (0%)	0 (0%)	5 (20%)	14 (75%)
I felt comfortable sharing my thoughts and ideas on Discord	1(5%)	2 (10%)	0 (0%)	3 (15%)	14 (70%)
I felt like the research team listened to my suggestions for improving SHER	1 (5%)	0 (0%)	1 (5%)	1 (5%)	17 (85%)
I feel like my participation has improved the SHER intervention	1 (5%)	1 (5%)	0 (0%)	7 (35%)	11 (55%)
I felt like I was able share all of my suggestions for improving SHER	1 (5%)	0 (0%)	1 (5%)	4 (20%)	14 (70%)
I feel comfortable visiting Discord to view or seek new information	1 (5%)	0 (0%)	3 (15%)	4 (20%)	12 (60%)
I feel comfortable posting my opinions on Discord to share knowledge	1 (5%)	1 (5%)	2 (10%)	5 (25%)	11 (55%)
